# Die Entwicklungsbeschleunigung von Robotic Process Automation Lösungen – Fallstudie einer deutschen Gesundheitsbehörde

**DOI:** 10.1365/s40702-021-00764-6

**Published:** 2021-08-02

**Authors:** Carolin Vollenberg, Julian Koch, Michael Trampler, Friederike-Maria Bade, André Coners, Ralf Plattfaut

**Affiliations:** 1 Fachbereich Technische Betriebswirtschaft, Fachhochschule Südwestfalen, Hagen, NRW Deutschland; 2 Fachbereich Elektrische Energietechnik, Fachhochschule Südwestfalen, Soest, NRW Deutschland

**Keywords:** Robotic Process Automation, Skalierung von IT-Systemen, Heterogene Systemlandschaft, Softwareverteilung, Aktionsforschung, Schnelle Anwendungsentwicklung, Robotic process automation, Scaling of IT systems, Heterogeneous system landscape, Software distribution, Action research, Fast application development

## Abstract

Krisen erfordern flexibles Handeln und schnelle Anpassungen sowohl von Menschen als auch von öffentlichen Institutionen. Die öffentliche Hand und insbesondere die deutschen Gesundheitsbehörden sind während der Covid-19-Pandemie, beginnend im März 2020, massiv herausgefordert. Die Bewältigung des teilweise exponentiell wachsenden Prozessvolumens durch kurzfristige Infektionsereignisse muss in kürzester Zeit fachgerecht erfolgen und unterliegt einer permanenten Reaktion und Anpassung an sich ändernde Rahmenbedingungen. Bestehende Strukturen und ineffiziente Prozesse erschweren diese notwendige Skalierung der Bearbeitung zunehmend. In diesem Beitrag werden mittels Aktionsforschung Ansätze für eine beschleunigte und flexible Entwicklung von Robotic Process Automation (RPA)-Lösungen zur Automatisierung bestehender Verwaltungsprozesse innerhalb einer betroffenen Gesundheitsbehörde in Deutschland untersucht. Als Ergebnis wurden Ansätze für eine schnellere und weniger komplexe Entwicklung von RPA-Lösungen in einer sehr schnell skalierenden und gleichzeitig stark heterogenen IT-Systemlandschaft erarbeitet und bewertet.

## Abstract (en)

Crises require flexible action and quick adjustments from both people and public institutions. The public sector and especially the German health authorities are massively challenged by the Covid-19 pandemic, starting in March 2020. The management of the partially exponentially growing process volumes due to short-term infection (superspreading) events must be carried out professionally in the shortest time possible and is subject to permanent reaction and adaptation to changing conditions. Existing structures and inefficient processes make this necessary scaling of processing increasingly difficult. In this article, approaches for an accelerated and flexible development of Robotic Process Automation (RPA) solutions for the automation of existing administrative processes within an affected health authority in Germany are investigated by means of action research. As a result, approaches for a faster and less complex development of RPA solutions in a very fast scaling and at the same time heterogeneous IT system landscape were elaborated and evaluated.

## Einleitung

Die Wichtigkeit der Digitalisierung und Modernisierung von Verwaltungsprozessen im öffentlichen Gesundheitssektor steigt rasant. In der Krisensituation der Pandemie, die in Deutschland im März 2020 begann, wird diese notwendige Anpassung noch einmal präsenter und gibt einen zusätzlichen Impuls. Die Beteiligten werden mit der bisher ausgebliebenen Digitalisierung konfrontiert. Es müssen schnelle Reaktionen erfolgen. Diese erfordern von den öffentlichen Verwaltungen und Gesundheitsbehörden eine kurzfristige Realisierung und Umsetzung neuer Aufgaben und Prozesse (Hoppe et al. 2020). Dabei erfordert die Pandemie von der öffentlichen Hand flexibles und vor allem reaktionsschnelles Handeln. In dieser Zeit wird besonders deutlich, wie wichtig ein ganzheitlich funktionierendes öffentliches System ist. Bei der Erfassung und Nachverfolgung von akuten Krankheitsfällen stoßen die kommunalen Verwaltungen, insbesondere die Gesundheitsbehörden, zunehmend an ihre Kapazitäts- und Belastungsgrenzen. Zusätzlich stellen sich die fehlende informationstechnische Aufbereitung und der Einsatz uneinheitlicher elektronischer Systeme als Herausforderungen dar und erschweren die Arbeit der beteiligten Verwaltungen (Piwernetz und Neugebauer 2020). Häufig wird weiterhin mit Zettel und Stift gearbeitet und Fallzahlen sowie Testergebnisse müssen händisch, teilweise mit Faxgeräten, weitergeleitet werden (Hoppe et al. 2020). Die aktuelle Pandemie zeigt, dass vor allem die öffentlichen Verwaltungen schnell handeln müssen, um ihre Arbeitsfähigkeit zu sichern. Dies beschleunigt wiederum die Digitalisierung (Piwernetz und Neugebauer 2020). Was zuvor ein eher behäbiger Entwicklungsprozess war, hat nun an Dynamik gewonnen. Die Erfahrungen mit virtuellen Konferenzen, Webinaren, leichtgewichtigen IT-Tools usw. ermutigen viele Verwaltungsmitarbeiter, selbst neue Methoden zu nutzen, um ihre eigenen digitalen Kompetenzen auf- und auszubauen. Aktuelle Untersuchungen zeigen auch, dass sich der durch die Pandemie erzeugte Druck positiv auf die Experimentier- und Veränderungsbereitschaft der öffentlichen Verwaltungen auswirkt.

Dies zeigt aber auch, dass die disparaten IT-Infrastrukturen in den öffentlichen Verwaltungen es erschweren, schnell zu reagieren und flexibel und proaktiv zu agieren. Aufgrund des in Krisenzeiten erhöhten Prozessvolumens werden die Personalkapazitäten zunehmend erweitert und neue Mitarbeiter werden eingestellt. Dieses erforderliche aber enorme Wachstum an Mitarbeitern und das damit verbundene hohe Aufkommen neuer IT-Systeme steigert die heterogenen Strukturen in den öffentlichen Verwaltungen. Hinzu kommt der Einsatz von ständig wechselnden verwaltungsfremden Mitarbeitern, die unter anderem aus dem Ruhestand oder der Bundeswehr hinzu kommen (Asatiani und Penttinen 2016). Der Einsatz von alten Laptops mit unterschiedlichen Betriebssystemen, veralteten Ordnerstrukturen, variablen Laufwerksstrukturen und die Ausstattung der Mitarbeiter mit neuen Laptops führt zu einer Vielzahl von Problemen und verhindert eine stabile, effiziente und sichere Prozessabwicklung. Neben den heterogenen Systemen arbeiten die Mitarbeiter in unterschiedlichen Verwaltungsgebäuden und im Homeoffice. Die gezwungenermaßen schnell wachsenden IT-Infrastrukturen, das stark abweichende Know-How der neuen Mitarbeiter und das zusätzlich fehlende IT-Infrastukturmanagement in öffentlichen Verwaltungen begünstigen diese Heterogenität weiter und machen eine ganzheitliche Homogenisierung aller Systeme und der gesamten IT-Infrastruktur innerhalb kürzester Zeit unmöglich. Im Gegensatz dazu können sich Organisationen, die zu Beginn der Krise über die entsprechende technische Ausstattung, IT-Infrastruktur und Personal verfügten, schnell auf die neuen Gegebenheiten einstellen und die plötzliche hohe Prozessbelastung zum Teil mit technischen Hilfsmitteln kompensieren (Fricke 2021).

Daraus ergibt sich auf der Forschungsseite ein Begleitbedarf für die Entwicklungsprozesse von Automatisierungslösungen, die in kürzester Zeit direkt auf die Pandemie-Situation skaliert werden müssen. Die Pandemie erfordert hierbei schnelle Reaktionen, so dass der Druck zur Implementierung neuer Prozesse steigt. Die hier erforschte Technologie der Robotic Process Automation (RPA) bringt an dieser Stelle die entsprechenden Voraussetzungen für eine schnelle Automatisierung von Unternehmensprozessen mit und ist somit geeignet für eine entsprechende Entlastung der Mitarbeiter zu sorgen (Asatiani und Penttinen 2016).

Dieser Artikel fokussiert anhand von Aktionsforschung im öffentlichen Sektor zu Krisenzeiten (hier: Pandemie) die Ableitung notwendiger Ansätze, um RPA-Lösungen ad-hoc und mit sehr geringem Aufwand zu entwickeln. Dabei können wir anhand der Automatisierung verschiedener IT-basierter Verwaltungsprozesse aufzeigen, welche Formen der Aufgabenteilung und Kombinationen aus RPA-Lösung mit menschlicher Assistenz personelle Engpässe sinnvoll kompensieren und die Effizienz und Qualität eines Prozesses nicht nur steigern, sondern ihn auch in kürzester Zeit krisensicher machen können.

Zusammengefasst wird damit eine Hauptforschungsfrage angesprochen: *„Wie können RPA-Lösungen unter hohem Zeitdruck und mit sehr geringem Zeitaufwand qualitativ ausreichend entwickelt werden?“*

Der weitere Verlauf dieses Artikels ist wie folgt gegliedert. Zuerst stellen wir die aktuelle Situation der rapide gewachsenen Prozess- und IT-Landschaft innerhalb der Gesundheitsbehörden in Zeiten der Pandemie dar und erläutern die Technologie der RPA. Anschließend erläutern wir die angewandte dreiteilige Aktionsforschungsmethodik und stellen die Datenquellen und das Verfahren der Datenerhebung vor. Der vierte Abschnitt enthält die Ergebnisse unserer Aktionsforschung. Wir schließen den Artikel in Abschnitt fünf mit einer Zusammenfassung unserer Forschung, in der wir die Limitationen unserer Forschung sowie die Implikationen für die Praxis und Theorie ansprechen.

## Hintergrund

Aufgrund der Notwendigkeit der kontinuierlichen Verfolgung von Infektionsketten führt die Pandemie zu einem exponentiellen Anstieg des bürokratischen Aufwands innerhalb der kommunalen Gesundheitsbehörden (Steppat 2021; Karl 2020). Mit dem Beginn der Lockerung der weitreichenden Kontaktverbote im April 2020 wird die Sicherstellung einer flächendeckenden Kontaktverfolgung durch die Gesundheitsbehörden von zentraler Bedeutung. Das Bundesministerium für Gesundheit hat am 1. Mai 2020 ein Konzept und einen Umsetzungsplan zur Kontaktverfolgung vorgelegt, der die Grundlage für das Handeln in den Kommunen bildet (Korzillus 2020). Die geforderte Dokumentation von Quarantänefällen und die daraus resultierenden wiederkehrenden Anfragen von Patienten und potenziellen Patienten belasten die Gesundheitsbehörden zunehmend und führen zu einem hohen Prozessaufkommen und steigenden Prozessaufwänden, die in der Summe manuell nicht zu bewältigen sind (Korzillus 2020). Bei einer gleichzeitigen raschen Aufstockung der Ressourcen ist dies jedoch problematisch. Neue Prozesse müssen parallel zu den bestehenden verwaltet werden, und zwar zu einem großen Teil von neuen und fachfremden Mitarbeitern (Zimmermann 2021). Hinzu kommen die Komplexität und die Vielfalt der Regelungen, welche technischen Hilfsmittel, insbesondere Software, eingesetzt werden dürfen und welche nicht. Zugleich sind viele Verwaltungen sequenziell-hierarchisch organisiert, was Sprunginnovationen verhindert und die Effektivität reduziert (Korzillus 2020).

Prozessautomatisierung stellt eine bewährte Methode zur Optimierung von Abläufen dar, um die Effizienz und Effektivität von Unternehmen und Prozessen zu steigern (Plattfaut 2019). Eine Möglichkeit der Prozessautomatisierung, die in der untersuchten Kommune bereits vor der Pandemie in geringem Umfang genutzt wurde, ist der Einsatz von RPA (Asatiani und Penttinen 2016).

Bei RPA handelt es sich um Software-Roboter, die es ermöglichen, repetitive und regelbasierte Aufgaben zu automatisieren (Syed et al. 2020). Typischerweise findet RPA Anwendung in Prozessen, in denen die Mitarbeiter vor einer Prozessautomatisierung beispielsweise Daten aus unterschiedlichen digitalen oder analogen Medien entgegennehmen, diese verarbeiten, um sie anschließend in bestehende Systeme einzugeben (Willcocks et al. 2015). RPA imitiert die menschlichen Interaktionen auf den Bedieneroberflächen der IT-Systeme und automatisiert die Dateneingabe und den Datenaustausch systemübergreifend (Syed et al. 2020). RPA verfolgt das Ziel „in konstanter Art und Weise bessere Resultate mit weniger Aufwand zu erbringen“ (D’Onofrio und Meinhardt 2020). Mithilfe von RPA wird die flexible Anpassung an Kapazitätsbedarfe und Arbeitsbelastungen angestrebt (Plattfaut 2019). Diese Technologie kann dabei vergleichsweise niedrigschwellige, schnell zu implementierende und kostengünstige Lösungen bieten (Syed et al. 2020). Durch die Vorteile von RPA – Kosteneinsparungen, Schnelligkeit, Erhöhung von Genauigkeit bei der Ausführung von wiederkehrenden Prozessen – werden Mitarbeiter entlastet, Fehlerquellen reduziert und mögliche Geschäftsverluste kompensiert (Koch et al. 2020). Darüber hinaus können plötzliche Bedarfsschwankungen, die seitens der Pandemie durch unvorhersehbare Maßnahmen auftreten, mittels einer hohen Skalierbarkeit von RPA abgefangen werden (Plattfaut 2019). Administrative Aufgaben werden schneller, genauer und unermüdlicher erledigt (Koch et al. 2020).

RPA wird in vielen Anwendungsbereichen zunehmend vertieft wissenschaftlich untersucht und ist Gegenstand zahlreicher Fallstudien, zum Beispiel im öffentlichen Sektor (Koch et al. 2020), Bildung (Herbert 2016), Finanzindustrie (Willcocks et al. 2017), Telekommunikation (Lacity et al. 2015), Recht (Herbert 2016), Personalwesen (Hallikainen et al. 2018) oder IT (Plattfaut et al. 2020). Trotzdem besteht weiterhin Forschungsbedarf in Bezug auf Faktoren, die die technische RPA-Implementierung in diversen Kontexten beschleunigt (Syed et al. 2020).

In der Literatur existieren verschiedene Kriterien, die das Automatisierungspotential von Geschäftsprozessen durch RPA bewerten. Prozesse mit geringem kognitivem Anteil, hohem Anteil an Routineaufgaben und vergleichsweise hohem Personaleinsatz bieten Potenzial zur Automatisierung mithilfe von RPA (Asatiani und Penttinen 2016). Sogenannte Hintergrundprozesse, also IT-gestützte Bearbeitungsprozesse, die für den Mitarbeiter mit monotoner, repetitiver Arbeit verbunden sind, werden häufig mit Hilfe von RPA abgebildet (Syed et al. 2020). Diese Hintergrundprozesse sind im öffentlichen Sektor in großem Umfang vorzufinden (Balka et al. 2018): Eingabemasken ausfüllen, Auslesen und Aktualisieren von Einträgen in Datenbanken und Datenextraktionen aus Formularen sind nur einige Beispiele, die einen relativ großen Anteil der Arbeiten in der öffentlichen Verwaltung ausmachen. So könnten in den deutschen Behörden bis zu 62 % aller Arbeitsstunden automatisiert ablaufen (Balka et al. 2018). Im aktuellen wissenschaftlichen Diskurs, bezogen auf unseren Fallkontext, werden die Anwendungsmöglichkeiten und die Vorteile von RPA als zeit- und ressourcensparende Automatisierung von Geschäftsprozessen dargestellt, um Mitarbeiterausfälle bei unvorhersehbaren Umstrukturierungen und Verläufen zu kompensieren (Lacity et al. 2015; Syed et al. 2020).

## Methode

Die gewählte Methode für die vorliegende Forschungsarbeit ist die Aktionsforschung. Aktionsforschung ist ein Ansatz, bei dem der Forscher und Mitglieder der Organisation zusammenarbeiten, um Probleme zu diagnostizieren und zu lösen. In diesem Artikel wird die Aktionsforschung verwendet, um ein Phänomen zu beschreiben und zu analysieren, während der Forscher selbst beteiligt ist und die Eigenschaften beeinflusst (Susman and Evered 1978; Davison et al. 2004). Ein Hauptmerkmal der Aktionsforschung ist es, dass Wissen nicht nur durch Beobachten und Analysieren, sondern auch durch Beeinflussen und Mitwirken gewonnen wird (Baskerville und Wood-Harper 1996; Coughlan und Coghlan 2002; Coghlan 2011). Dieser dynamische Ansatz erlaubt der Forschung, sich flexibel von den Bedürfnissen der Mitarbeiter und der Arbeitsumgebung leiten zu lassen. Zudem bietet dieser reaktionsfähige Ansatz die Möglichkeit, dynamische und ad-hoc geforderte Änderungen in Prozessen reaktionsschnell zu realisieren. Die Relevanz und der Bedarf an Aktionsforschung ist forschungsseitig durch die sehr volatilen und hohen Prozessmengen sowie manuellen Prozessaufwände in dieser Forschungsarbeit definiert.

Wir wenden das Konzept der Aktionsforschung an, um die Anwendbarkeit von RPA im öffentlichen Sektor zu analysieren. Während wir die Mitarbeiter in RPA ausbilden und die Methoden und Werkzeuge verbreiten, beeinflussen wir den Fortschritt der RPA-Entwicklung. Die Autoren begleiten dabei die RPA-Entwicklung aktiv und sind in das Projektteam integriert. Diese generieren sowohl eigene Lösungen als auch gemeinsame Lösungen mit den Mitarbeitern im Entwicklerteam. Der Entwicklungsprozess ist anhand von verschiedenen Dokumentationen wie die RPA-Ausführungsprotokolle, Projektstatusberichte, Entwicklungshistorien der RPA-Lösungen und Projektdokumentationen aufgezeichnet. Die Datenerfassung spiegelt die Metaphase der Aktionsforschung wider und wird kontinuierlich innerhalb der Forschungszyklen realisiert. Sobald eine RPA-Lösung für einen Prozess vorliegt, wird der Entwicklungsprozess durch Interviews mit den Mitarbeitern reflektiert und die Datenbasis ergänzt. Die vorhandenen Dokumentationen werden dann einer qualitativen Inhaltsanalyse unterzogen (Mayring 2004).

Die RPA-Ausführungsprotokolle, die standardmäßig von der Entwicklungsplattform während der Ausführung von RPA generiert werden, werden hinsichtlich der verwendeten Erweiterungsimplementierungen (z. B. Plug-ins), Laufzeiten und Fehlertypen, Ausnahmebehandlung, Absprünge oder anderer Ausführungsstopps analysiert und bewertet. Auf diese Weise werden deutlich quantifizierbare Qualitätsmetriken gewonnen, die erste objektivierbare Einblicke in das jeweilige Entstehen von RPA-Lösungen und mögliche Entwicklungsbarrieren geben.

Die Projektstatusberichte werden mit diesen oben genannten Erkenntnissen auf der Ebene der Entwicklungszyklen analysiert, um mehr Kontext über die Inhalte und mögliche Verbesserungen oder Umstrukturierungen der jeweiligen RPA-Lösung, auch im Hinblick auf die betrachteten RPA-Ausführungsprotokolle, zu erhalten.

Die Entwicklungshistorien der RPA-Lösungen werden zudem, ergänzend herangezogen, um die Veränderung der Entwicklungsqualität besser nachvollziehen zu können und so eine etwas detailliertere Aussage über die Überarbeitungen, Ergänzungen und Umstrukturierungen der RPA-Lösungen zu erhalten. Die so erstellte Historie wird auch genutzt, um erste Lernkurven nachzuzeichnen und so den Einfluss und die Wirkung bestimmter Entwicklungsparadigmen von RPA besser zu verstehen.

**Abb. 1:**
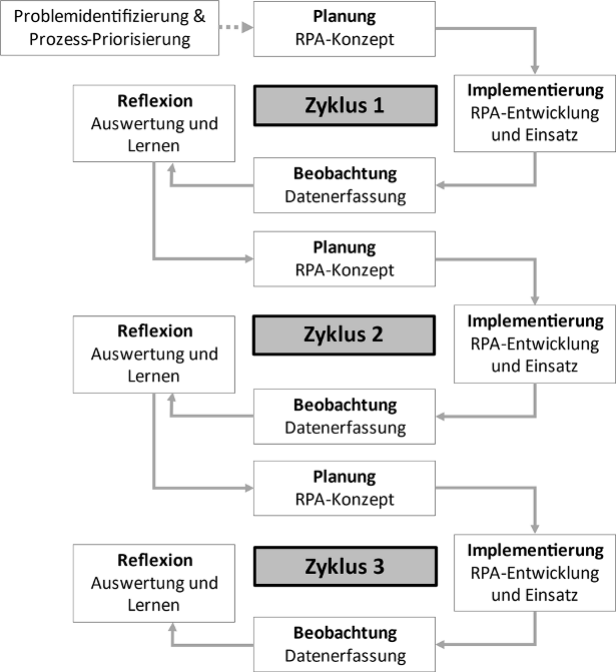
Aktionsforschung in Anlehnung an Davison et al. (2012)

Im Rahmen von gemeinsamen Workshops werden diese oben genannten Erkenntnisse in den Entwicklungsprozess eingearbeitet. Der Entwicklungsprozess der RPA-Lösungen wird anhand von drei Zyklen, die jeweils die Hauptphase der Aktionsforschung darstellen, realisiert (vgl. Abb. 1).

Konkret durchlaufen wir die in Abb. 1 dargestellten Entwicklungszyklen für die Automatisierung mithilfe von RPA wie folgt:

Zunächst werden die Probleme identifiziert und die zu automatisierenden Prozesse priorisiert. Anschließend folgt im *ersten Zyklus* die Konzepterstellung. Darauf folgt die Entwicklung einer jeweiligen RPA-Lösung durch Mitarbeiter in Zusammenarbeit mit den Autoren. Dabei werden Daten in Form von Interviews mit Mitarbeitern, Projektdokumentationen aus den Projektstatusberichten und RPA-Ausführungsprotokollen aus der RPA-Entwicklungsumgebung zusammengetragen (vgl. Abschn. 3.2). Identifizierte Fehler während der Entwicklung sowie Fehler der RPA-Lösung im Einsatz werden in drei Fehlerarten eingeteilt. Anschließend folgt eine gemeinsame Reflexion mit Feedbackschleifen zur RPA-Entwicklung und dem Einsatz der RPA-Lösung sowie die Auswertung aller Daten.

Im *zweiten Zyklus *arbeiten die Autoren mit einem festen Projektteam der Gesundheitsbehörde an der Konzepterstellung, in der RPA-Entwicklung und dem RPA-Einsatz sowie der Datenerfassung und -auswertung. Anschließend erfolgt eine vertiefte Datenerhebung über die Entwicklung und den Einsatz der RPA-Lösung. Daten über die vorhandenen und korrigierten Fehlerarten werden erneut gesammelt und verglichen, um eine Lösungsbewertung durchzuführen. Analog zum vorherigen Zyklus folgen Feedbackschleifen und Datenanalysen in Form von Interviews, Auswertung von Projektstatusberichten und RPA-Ausführungsprotokollen zur RPA-Lösung.

Im *dritten Zyklus* erfolgt die Vorgehensweise analog zu den vorherigen Entwicklungszyklen. Die Mitarbeiter überführen die Schwachstellen und Fehlerarten in das Konzept und verbessern die RPA-Lösung. Der Einfluss der Autoren beschränkt sich auf Schwachstellen in der Performance der RPA-Lösung.

### Teilnehmer und Fallbeschreibung

Die in diesem Artikel untersuchte Kommune ist eine Großstadt in Westdeutschland mit etwa 200.000 Einwohnern. Diese weist seit Beginn der Pandemie sehr hohe Inzidenzraten und Infektionen auf und wurde demnach kontinuierlich als Hochrisikogebiet eingestuft. Die Gesamtzahl der Mitarbeiter der Gesundheitsbehörde betrug 64, davon 39 in Vollzeit und 25 in Teilzeit. Als Einrichtung des öffentlichen Gesundheitsdienstes sind die Aufgaben der untersuchten Gesundheitsbehörde in erster Linie durch das Gesetz über den öffentlichen Gesundheitsdienst definiert. Vorrangiges Ziel ist die Daseinsvorsorge und, als ein unverzichtbarer Dienstleister, die Gesundheit der Bevölkerung zu fördern und zu schützen (Reisig und Kuhn 2016).

Zwei Autoren arbeiteten bereits vor der Pandemie in anderen Kontexten erfolgreich mit der öffentlichen Verwaltung an der Prozessoptimierung und Prozessautomatisierung und wurden explizit um Unterstützung während der Krise gebeten. Die hier dargestellte Gesundheitsbehörde ist von den Entscheidungsträgern der öffentlichen Verwaltung und dem Autorenteam auch deshalb ausgewählt worden, weil viele Mitarbeiter Schwierigkeiten haben, belastbare Prozesse auszuführen oder auf das deutlich gestiegene Prozessvolumen sowie die dynamischen Prozessänderungen während der Pandemie zu reagieren. Personalkapazitäten zur Bewältigung bestehender sowie neuer Prozesse und Aufgaben, die durch die Pandemie eingeführt werden, reichen nicht mehr aus. Daher werden zum Teil neue, teilweise fachfremde Mitarbeiter eingestellt. Zusätzlich weist diese Kommune eine hohe Heterogenität in den bestehenden IT-Systemen auf.

Die Teilnehmer dieser Aktionforschung sind Sachbearbeiter (*n* = 6) in der untersuchten Gesundheitbehörde in der Abteilung für Infektionsschutz, die speziell als Reaktion auf die Pandemie geschaffen wurde. Keiner von ihnen hat zuvor Erfahrungen mit der RPA-Technologie oder sonstiger Prozessautomatisierungstechnologien gemacht. Zudem beteiligen sich die Autoren ergänzend durch Begleitung der Teilnehmer bei der selbstständigen Entwicklung von RPA-Lösungen.

In dem vorliegenden Artikel werden RPA-Lösungen für die IT-gestützten Verwaltungsprozesse der Gesundheitsbehörde untersucht. In Summe sind dies 42 Subprozesse, die aufgrund der Krisensituation neu implementiert werden, sowie 13 bestehende Verwaltungsprozesse, die bisher manuell durch händische Dokumentation umgesetzt werden. Wir haben in diesem Artikel einen Subprozess immer als einen Teil eines darüber liegenden komplexen Prozesses definiert, der logisch abgeschlossen ist und von einem anderen Prozess oder Subprozess aufgerufen werden kann. Ein Beispiel für einen Subprozess ist die Extraktion und Transformation von Personenstammdaten aus einer Datenbankanwendung in eine Excel-Datei und die anschließende regelbasierte Archivierung und Kennzeichnung dieser Datei. Diese Prozesse lassen sich übergeordnet in diesem Artikel durch das Neuanlegen von Personen, die Pflege von Ansprechpartnern sowie die Eingabe und Aktualisierung der zugehörigen Adressdaten spezifizieren. Des Weiteren fokussieren sich diese neuen Verwaltungsprozesse auf die Pflege von Kontakthierarchien und Kontaktbeziehungen zur Sicherstellung einer adäquaten Nachverfolgung sowie den Import, Export und Abgleich von Daten aus Portalen der Behörde.

### Datenerfassung

Die Datenerhebung erfolgt durch drei Datenquellen während und parallel zu den Aktionsforschungszyklen, die sowohl die Entwicklung der RPA-Lösungen (Projektstatusberichte und Entwicklunghistorien) als auch die Bewertung der Lösungen (durch Interviews mit Mitarbeitern und RPA-Ausführungsprotokolle) integrieren. Dazu haben wir im ersten Entwicklungszyklus zunächst drei Fehlerarten in Zusammenarbeit mit den Mitarbeitern und durch die Analyse der RPA-Ausführungsprotokolle, Projektstatusberichte und Entwicklungshistorien identifiziert, die zum einen zu Problemen bei der Ausführung der RPA-Lösung führen und den Prozessablauf verhindern. Zum anderen werden Fehler identifiziert, die aus Sicht der Mitarbeiter zu einem Problem bei der Ausführung der RPA-Lösung werden können oder eine schnellere RPA-Lösungsentwicklung generell verhindern werden:

- *Reale Fehler*: Tatsächliche Probleme, die die Entwicklung oder Ausführung einer RPA-Lösung verhindern
- *Emergente Fehler:* Performance- oder Skalierungsprobleme bei der RPA-Lösung, wobei aber die Ausführung funktioniert
- *Testfehler*: Hypothetische Fehler, die zu einem Problem bei der RPA-Lösung führen können

Bezogen auf diese drei möglichen Fehlerarten werden in den oben beschriebenen Aktionsforschungszyklen kontinuierlich Fehlerdaten gesammelt, analysiert und vor diesem Hintergrund neue Konzepte und RPA-Lösungen geplant, umgesetzt und evaluiert.

Die Entwicklung der RPA-Lösung wird im jeweiligen Zyklus zunächst geplant. Der zu automatisierende Prozess wird anhand der im Abschn. 3.1 dargestellten Mitarbeiter als realer, emergenter und hypothetischer Fehler identifiziert, notwendige Konfigurationen, Software und Datenquellen werden ermittelt. Anschließend erfolgt die Entwicklung der jeweiligen RPA-Lösungen zur Automatisierung. Die jeweilige Entwicklung wird anschließend in der praktischen Anwendung analysiert und bewertet. Am Ende jedes Entwicklungszyklus werden die Teilnehmer erneut in einem halbstrukturieren Interview befragt. Zu diesem Zweck haben wir die Inhalte der RPA-Ausführungsprotokolle ergänzend analysiert. Die RPA-Ausführungsprotokolle werden standardmäßig innerhalb der RPA-Entwicklungsumgebung erzeugt und lassen Rückschlüsse auf bestimmte Entwicklungsprobleme und -fortschritte zu. Diese Erkenntnisse können wir in die Befragung der Teilnehmer (*n* = 6) mit einbeziehen. Konkret bitten wir die Teilnehmer in den halbstrukturierten Interviews (39 Interviews mit einer Gesamtdauer von 24,9 h), die RPA-Einsätze sowie die Entwicklungsansätze dieser zu beschreiben, ihr Vertrauen in die Belastbarkeit der Ansätze, die zur Beschleunigung solcher Einsätze verwendet werden sowie ihr Vertrauen in die Lösung von Beschleunigungsproblemen verschiedener Art. Die Fragen im Interview befassen sich mit den Eindrücken der Mitarbeiter vom beschleunigten RPA-Entwicklungs-Ansatz, ihrer wahrgenommenen Nützlichkeit für ihre zukünftigen Einsatzanforderungen und ihrem allgemeinen Vertrauen in die Technologie. Die Interviews werden in den Diskussionskanälen jeweils am Ende des Forschungszyklus in der Reflexion (29) und unmittelbar nach der Implementierungsphase (8) und immer im Vorfeld des nächsten Entwicklungszyklus (2) geführt. Mit Ausnahme des ersten Forschungszyklus werden die Interviewthemen dabei auf Basis der gesammelten Fehlerdaten und der Dokumentation der RPA-Entwicklung (bestehend aus Projektstatusberichten, RPA-Ausführungsprotokollen, Entwicklungshistorien) zusammengestellt und vom Forschungsteam ausgearbeitet. Durch das Reflektieren der Ergebnisse können die gemachten Erfahrungen der Teilnehmer im nächsten Zyklus aufgenommen und eingearbeitet werden. Dadurch wird sowohl der Entwicklungsansatz als auch die RPA-Lösung sukzessive und kontinuierlich verfeinert. Zum direkten Vergleich der qualitativen Erhebungen werden zusätzlich Projektstatusberichte gesammelt, die während des jeweiligen Entwicklungszyklus generiert und in Form einer Dokumentenanalyse ausgewertet werden. Die Ergebnisse werden mit den Aussagen aus den jeweiligen Interviews verglichen und dienen ergänzend dazu, um missverständliche Zusammenhänge zu identifizieren und weitere Fragen für nachfolgende Befragungsrunden zu entwickeln.

Nach jeder Datenerhebung stellen die Autoren die Antworten zusammen und entscheiden gemeinsam mit den Mitarbeitern, wie mit jedem RPA-Entwicklungsschritt bis zur nächsten Datenerhebung verfahren werden soll. Es erfolgt im nächsten Zyklus eine erneute Planung des Entwicklungsprozesses, die sich jeweils auf die am Ende des vorherigen Zyklus durchgeführten Reflektion bezieht.

### Datenanalyse

Die Daten werden sowohl *formativ* (um detaillierte Informationen über spezifische Änderungen im Entwicklungsansatz als direkte oder indirekte Auswirkung auf die RPA-Lösung zu erhalten) als auch *summativ* (als Vergleich zwischen postuliertem und erreichtem Sollzustand der RPA-Lösung) erhoben (Petersen et al. 2014). In Übereinstimmung mit den Praktiken der Aktionsforschung treffen sich die Autoren nach jedem der Datenerhebungsereignisse (sowohl Entwicklungsereignisse als auch Interviews), um in einem fortlaufenden Analyseprozess aufkommende Erkenntnisse und Änderungen für den nächsten Forschungszyklus zu diskutieren.

Ein Teil des Autorenteams führt eine offene Kodierung der Mitarbeiterbefragungen (der halbstrukturierten Interviews) durch, um deren Nutzung der evaluierten RPA-Lösungen und ihr Verständnis der beschleunigten Entwicklungsschritte zu erfassen. Die Triangulation über mehrere Datenquellen, insbesondere über die Interviews, die Projektstatusberichte, die Entwicklungshistorien der RPA-Lösungen und die RPA-Ausführungsprotokolle, unterstützt, die Validität sicherzustellen und Bereiche mit Übereinstimmungen oder Konflikten zu identifizieren. Insbesondere durch die Projektstatusberichte und RPA-Ausführungsprotokolle, die den Reifegrad der RPA-Lösung ausmachen, können wir während des gesamten Forschungprozesses Daten sammeln, untersuchen und reflektieren. Als Teil des Entwicklungsteams treffen sich die Autoren regelmäßig (alle 3–6 Tage) mit den teilnehmenden Mitgliedern des Entwicklungsteams, um mögliche Diskrepanzen und Probleme in den Entwicklungsansätzen der RPA-Lösung zu besprechen, bis ein Konsens über den Entwicklungsfortschritt und die Implementierungshindernisse erreicht ist.

## Ergebnisse

Anhand der dargestellten Aktionsforschung im öffentlichen Sektor in einer Krisensituation (hier: Pandemie) können wir Lösungsansätze ableiten, die eine schnelle Entwicklung von RPA-Lösungen fördern.

Vorerst betrachten wir die Ergebnisse aus den einzelnen Entwicklungszyklen (vgl. Abb. 1). Nach dem ersten *Zyklus* sind bereits Schwachstellen sowie Fehlerarten der RPA-Lösung identifiziert worden und Lösungsmöglichkeiten können auf Basis der eingeteilten Fehlerarten abgeleitet werden. Es wird deutlich, dass die Rahmenbedingungen für die RPA-Entwicklung und den spezifischen Einsatz der RPA-Lösung angepasst werden müssen. Durch das ständige Projektteam werden so bereits aufgetretene Fehler vermieden, was die Entwicklung in den weiteren Phasen erleichtert.

Im zweiten *Zyklus* können wir durch eine verbesserte Kommunikation innerhalb des Projektteams die bereits identifizierten und korrigierten Fehlerarten vergleichen. Somit führt die Lösungsbewertung zur Entwicklung einer besseren RPA-Lösung.

Im letzten (dritten) *Zyklus* ist die Vorgehensweise bereits bekannt und das Verständnis für die jeweiligen Entwicklungsphasen im Zyklus ist gefestigt. Die Mitarbeiter sind mit der Entwicklung der RPA-Lösung vertraut, sodass diese die Schwachstellen selbständig identifizieren. Durch die Anwendung in der Gesundheitsbehörde werden ausreichend Erfahrungen für die Übertragung von RPA-Lösungen auf andere Organisationseinheiten gesammelt. Im Ergebnis wird eine Sammlung von automatisierten Prozess-Inkrementen zusammengestellt, die allen Mitarbeitern zur Verfügung gestellt wird. Diese sind dadurch in der Lage, die benötigten komplexeren Prozessabläufe *„[…] schneller aus der Sammlung fertiger Prozessschritte*“ (Mitarbeiter 3) zusammenzustellen.

Durch das enorme Wachstum der Mitarbeiterzahl aufgrund der Pandemie und das damit verbundene Aufkommen an IT-Systemen ist in kurzer Zeit eine sehr große Anzahl an disparaten Einzelsystemen entstanden, die eine unübersichtliche und heterogene betriebliche IT-Systemlandschaft darstellen. Aufgrund dieser Unterschiede in den Voraussetzungen der Arbeitsplatzsysteme identifiziert unsere Forschung zwei Ansätze, um RPA-Lösungen unter hohem Zeitdruck mit geringem Zeitaufwand zu entwickeln. Die gemeinsame Erarbeitung und Entwicklung der RPA-Lösungen von und mit den Mitarbeitern unter Einbezug aller gemachten Erfahrungen beim Einsatz einer jeweiligen RPA-Lösung, der eruierten Fehlerdaten und der Dokumentationen (bestehend aus Projektstatusberichten, RPA-Ausführungsprotokollen, Entwicklungshistorien) lässt die Ableitung der nachfolgend dargestellten Lösungsansätze zu:

1. Einsatz einer RPA-Lösung als Übersetzungsschicht
2. Inkrementelle RPA-Entwicklung

### *1. RPA-Lösung als Übersetzungsschicht*

Der erste Ansatz beschreibt den Einsatz einer RPA-Lösung, die die Umgebungsvariablen des jeweiligen Betriebssystems erfasst und um eine Übersetzungsschicht ergänzt. Diese Übersetzungsschicht wird als eigenständige RPA-Lösung entwickelt und wirkt als notwendige Bedingung für den Einsatz aller anderen RPA-Lösungen. Mit Hilfe dieser RPA-basierten Übersetzungsschicht werden erste Fehlerquellen, wie inkonsistente Softwareversionen, unterschiedliche Ordnerstrukturen oder Laufwerksbenennungen, identifiziert. Die Übersetzungsschicht erzeugt dann eine Schicht mit einheitlichen Abhängigkeiten, Verknüpfungen und Konfigurationen für Betriebssystem-Umgebungsvariablen.

Die Schaffung einer Übersetzungsschicht zwischen dem Originalsystem und den eigentlichen RPA-Lösungen zur Automatisierung von Subprozessen hat den Effekt, dass eine schnellere Skalierung dieser RPA-Lösungen mit geringem Zeitaufwand möglich ist. Zum Teil werden *„[…] schwerwiegende Konflikte, Einrichtungsfehler und Systemabstürze vermieden“* (Mitarbeiter 1), so dass die jeweiligen RPA-Lösungen *„[…] ohne Hindernisse und ohne Nachbesserungen“* (Mitarbeiter 2) arbeiten können. *„Die Eigenständigkeit […]“* der RPA-Anwendung wird sichergestellt und *„[…] ein kontinuierliches Arbeiten ohne zeitlichen Ausfall unter hohem Zeitdruck“* (Mitarbeiter 1) gewährleistet.

**Abb. 2:**
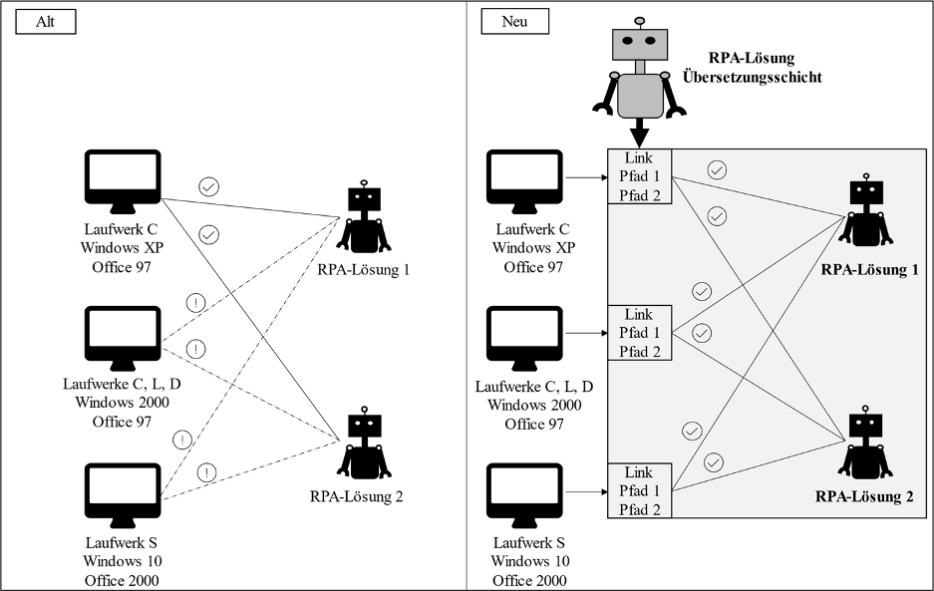
Vergleich der Entwicklungsansätze für RPA-Lösungen

Die folgende Abb. 2 visualisiert den Einsatz der RPA-Übersetzungsschicht. Die linke Seite spiegelt die Gegebenheiten in der heterogenen Systemlandschaft wider und verdeutlicht, dass eine Anwendung von RPA-Lösungen zur Automatisierung der Verwaltungsaufgaben nicht ohne ganzheitliche Systemumstrukturierungen funktionieren kann. Diese ganzheitlichen Umstrukturierungen und Anpassungen in der IT-Landschaft würden enorm viel Zeit in Anspruch nehmen. Daher verdeutlicht die rechte Seite den Benefit unseres Lösungsansatzes einer RPA-basierten Übersetzungsschicht zum Einsatz weiterer RPA-Lösungen. Durch die dort vorzufindende Übersetzungsschicht wird ein schneller und erfolgreicher Einsatz weiterer RPA-Lösungen ermöglicht.

Die weiterführende Entwicklung von RPA-Lösungen auf dieser Basis ermöglicht es, die verschiedenen RPA-Lösungen für die Managementprozesse in unterschiedlichen Systemumgebungen mit geringem oder gar keinem Änderungs- und Installationsaufwand schnell einzusetzen. Auf diese Weise wird eine valide und einheitliche Arbeitsumgebung generiert, auf der dann eine direkt lauffähige Entwicklung von RPA-Lösungen gewährleistet ist.

Der vorgestellte Lösungsansatz wird hier am Beispiel der RPA-basierten Erstellung einer Übersetzungsschicht für die vom Mitarbeiter verwendeten Systemvariablen dargestellt: Die RPA-Lösung führt zunächst einen Scandurchlauf der Microsoft Office-Version auf dem Betriebssystem des Mitarbeiters durch und unterscheidet zwischen den installierten Office-Versionen (Office 365, Office 2016, Office 2010), die unterschiedliche Installationspfade haben und auch unterschiedliche Anwendungsaufrufe innerhalb der Skriptsprache der RPA-Entwicklungsumgebung verwenden. Die RPA-Lösung legt eine einheitliche Sammlung von Verknüpfungen im Stammverzeichnis der jeweiligen RPA-Lösung an, über die die jeweilige Office-Anwendung, z. B. Excel, aufgerufen werden kann. Im nächsten Schritt werden die eingebundenen Netzlaufwerke hinsichtlich ihres gemappten Pfades bzw. Laufwerksbuchstabens standartisiert, so dass in diesem konkreten Beispiel auch Speicher- und Archivordner als Verknüpfungen im Wurzelverzeichnis der RPA-Lösung angelegt werden. Es gibt dabei zahlreiche solcher Anpassungsvorgänge der Systemumgebung, etwa für die Pfade der jeweiligen Verschlüsselungssoftware, Aufrufe der SharePoint-Komponenten oder des Dokumentenmanagements.

Bei dem vorgestellten Lösungsansatz ist anfangs mehr Aufwand in die Erfassung, Identifizierung und Abbildung der beschriebenen Fehlertypen, insbesondere der unterschiedlichen Umgebungsvariablen und Betriebssystemparameter, notwendig. Im weiteren Verlauf reduziert die RPA-basierte Übersetzungsschicht jedoch den Aufwand für Änderungs‑, Installations‑, Skalierungs- und Deployment-Prozesse auf anderen Systemen deutlich.

Dies ermöglicht mit Fortschreiten der Aktionsforschung einen schnelleren Entwicklungsprozess sowie qualitativ hochwertigere RPA-Lösungen (im Hinblick auf die Fehlerproduktion) und Anwendungen mit mehr Flexibilität (im Hinblick auf die Anpassungsfähigkeit) sowie weniger Ausfallzeiten (im Hinblick auf den Ressourcenverbrauch).

### *2. Inkrementelle RPA-Entwicklung*

Der zweite Ansatz zur beschleunigten Entwicklung von RPA-Lösungen beschreibt die inkrementelle Entwicklung von RPA-Lösungen und das Management dieser. Dieser Ansatz beinhaltet zum einen die bewusste Auswahl und Teilautomatisierung von Prozess-Inkrementen, also einzelner Subprozesse, sowie die gezielte Bereitstellung und der ausgewählte Einsatz dieser RPA-Lösungen.

Beim initialen Deployment von RPA-Lösungen auf dem Betriebssystem des Mitarbeiters werden die beschriebenen Systemanpassungsprozesse zunächst konsequent mit einer entsprechenden RPA-Lösung umgesetzt. Danach erst kann eine Priorisierung der zu entwickelnden RPA-Lösungen auf Subprozessebene vorgenommen werden. Aufbauend auf der RPA-basierten Übersetzungsschicht zur Systemvereinheitlichung werden anschließend Prozessautomatisierungen implementiert, die nach Fertigstellung zwischen den Endnutztersystemen kompatibel sind und somit auch zwischen Organisationseinheiten ausgetauscht werden können. Anstatt also einen Prozess in seiner Gesamtheit mittels RPA zu automatisieren, werden, wie zuvor erwähnt, die einzelnen Prozessabschnitte, also die Subprozesse, betrachtet und zur Automatisierung priorisiert. Wie zuvor bereits aufgegriffen wird unter anderem der Prozess zur Kontaktnachverfolgung in der Pandemie in seine einzelnen Subprozesse zerlegt. Der gesamte Prozess wird in die einzelnen Prozessabschnitte unterteilt. Insgesamt können 7 Subprozesse identifiziert werden. Diese lassen sich unterteilen in die Erstellung des Excel-Eingabeformulars, die Generierung der zentralen Datenbank, die Analyse des Bezirks, die Online-Datenabfrage, die Mitarbeiteranalyse, die Tagesberichte und die Quarantäne-Benachrichtigungen. Um die Informationen aus den generierten und analysierten Daten zu verwalten, muss RPA über verschiedene Schnittstellen und Graphical User Interfaces (GUI) kommunizieren.

Die imkrementelle RPA-Entwicklung und die Automatisierung eines Prozess-Inkrements lässt sich am Beispiel der Erstellung des Erfassungsbogens (als Prozess-Inkrement) im Kontaktnachverfolgungsprozess veranschaulichen und näher erläutern. Anstelle der durchgängig manuellen Datenerfassung wird der Erfassungsbogen mit Hilfe von RPA teilautomatisiert. Die Excel-basierten Erfassungsbögen werden von den Hotline-Mitarbeitern ausgefüllt und gespeichert. Am Ende des Arbeitstages verarbeitet die RPA-Lösung alle neu erstellten Erfassungsbögen und speichert deren Inhalte in einer Excel-Datenbank. Diese bildet die Grundlage für eine Datenauswertung (z. B. eine Bezirks- oder Mitarbeiteranalyse) und die Quarantänebescheide. Um die Fehleranfälligkeit dieses Prozesses bei der Dateneingabe zu verbessern, werden in der RPA-Lösung Kontrollregeln implementiert, die bestimmte Datenfelder (z. B. Postleitzahl) auf Plausibilität prüfen. Nach einem festen Zeitplan beginnt die RPA-Lösung, die neu erstellten Erfassungsformulare in ein strukturiertes Datenbankdokument zusammenzuführen. Dieses wird dann in das Dokumentenmanagementsystem der Behörde importiert und direkt in der entsprechenden Ordnerstruktur auf dem Netzlaufwerk abgelegt, im vorgegebenen Archivierungsformat verschlüsselt und mit einem Aufruflink und Metadaten wie dem Erstellungszeitpunkt auf der Intranet-Plattform angelegt. Der Subprozess zur Datenerfassung und -auslesung aus den Erfassungsbögen wird für die Automatisierung als Prozess-Inkrement aufgrund von hohen Aufwänden priorisiert und daher inkrementell eine RPA-Lösung entwickelt. Die zugehörige RPA-Lösung wird anschließend organisationsweit zur Verfügung gestellt.

Diese Teilautomatisierung der einzelnen Subprozesse in Form von Prozess-Inkrementen sowie die frei zur Verfügung stehenden RPA-Lösungen und dessen Management ermöglicht eine organisationsweite Nutzung dieser Ressourcen. Somit wird ein schnellerer Einsatz der jeweiligen RPA-Lösung bei unvorhersehbarem und plötzlichem Bedarf gewährleistet.

## Diskussion und Implikationen

Die Pandemie, die im März 2020 begann, ist unbestreitbar eine der größten Herausforderungen, mit denen die kommunalen Gesundheitsbehörden je konfrontiert werden. Die kurzfristige Aufstockung von Arbeitsplätzen und Personal ist durch die ad-hoc aufgetretene Notsituation in dem untersuchten Fallbeispiel enorm. Der Anpassungsdruck an steigende Prozessvolumina und die damit verbundene Notwendigkeit, neue IT-Infrastruktur bereitzustellen, führt zu einer weiteren Divergenz der eingesetzten Systemkonfigurationen in der ohnehin uneinheitlichen IT-Landschaft. Die vorgestellten Lösungsansätze können die Entwicklung von RPA-Lösungen mit sehr geringem Zeitaufwand gewährleisten. Das bedeutet, dass zunächst alle relevanten Umgebungsvariablen und Betriebssystemparameter auf allen Systemen mittels einer RPA-realisierten Übersetzungsschicht abgeglichen und harmonisiert werden. Anschließend gilt es relevante Prozess-Inkremente zu identifizieren und jeweilige RPA-Lösungen zu entwickeln, die durch die Übersetzungsschicht auf allen IT-Systemen und für jeden Mitarbeiter ad-hoc einsetzbar sind.

Mit den untersuchten RPA-Entwicklungsprozessen und den damit entwickelten zwei Lösungsansätzen, die einen solchen Prozess beschleunigen, kann sowohl die Projekteffizienz als auch die Eignung zur aktiven Beteiligung der Mitarbeiter an der RPA-Entwicklung eingehend analysiert werden. Der Fokus der Projektabwicklung in diesem Artikel liegt auf der effizienten und schnellen Fertigstellung der RPA-Lösungen unter Einhaltung aller prozessualen Anforderungen und unter besonderer Berücksichtigung aller durch Umgebungsbedingungen dynamisch ausgelösten Änderungen, insbesondere derjenigen, die spät im Entwicklungsprozess auftreten.

Das Foschungsdesign in diesem Artikel, ist eines, bei dem Praktiker und Forscher im Forschungsprozess zusammenarbeiten. Die Art und Weise, in der die hier genutzte Aktionsforschung die Entwicklung von integrativen Praktiken und Denkweisen erleichtert, bezieht sich auf drei miteinander verbundene Bereiche: Die Zusammenarbeit zwischen den Mitarbeitern der Gesundheitsbehörde, die reflektierende Praxis und die aktive Teilnahme der Forscher. Ähnlich dem theoretischen Modell von Davison et al. (2012), in dem die Reflexion mit dem Handeln in einem zweiseitigen Prozess verbunden ist, können wir in dieser Aktionsforschung sehen, wie die Mitarbeiter ihre Entwicklungsprozesse von RPA-Lösungen durch die Aktionsforschung reflektieren und sich entsprechend verändern. Gleichzeitig führen nicht nur die Zusammenarbeit der Mitarbeiter und die reflektierte Praxis zu Handlungen, sondern die aktive Teilnahme der Forscher ermöglicht auch eine weitere Reflexion und Veränderung der aktuellen Entwicklungspraktiken.

Allerdings sind vor allem in der Anfangsphase von Zyklus 2 Schwierigkeiten aufgetreten, valide Einstiegs- und Anknüpfungspunkte mit dem vorhandenen Mitarbeitern in dem besonderen volatilen Umfeld zu finden, da diese meist überlastet sind. Daher greifen die Abteilungsleitung und wir als Forscher, wie in den Reflexionsschleifen in Abb. 1 dargestellt, auf die Einrichtung eines regelmäßigen Kommunikations- und Diskussionskanals zurück, der feste Zeitfenster für die Mitarbeiter reserviert, um einen kontinuierlichen Austausch zu garantieren. Dies eröffnet den Mitarbeitern die Möglichkeit, ihr Wissen regelmäßig in einem festgelegten Raum auszutauschen und zu aktualisieren. Auf diese Weise können wir beobachten, wie sich das Wissen der Mitarbeiter festigt. Kombiniert mit der aktiven Teilnahme an den Entwicklungsprozessen der Anderen, können wir feststellen, dass sich das Wissen und die Fähigkeiten der Mitarbeiter im Bezug auf die RPA-Entwicklung und RPA im Allgemeinen verfestigt. Durch die Gestaltung der zeitlich begrenzten Termine erhalten die teilnehmenden Mitarbeiter nicht nur Antworten auf ihre eigenen Probleme und Fragen, sondern gewinnen auch ein neues Verständnis für die Probleme der anderen Mitarbeiter, die in diesen Terminen besprochen werden.

So können wir durch den Aktionsforschungsprozess die Arbeitsweisen und Entwicklungsprozesse hinter den RPA-Lösungen genauer beobachten und verstehen. Darüber hinaus ermöglicht der Aktionsforschungsprozess durch die offene Diskussion in den Reflexionsschleifen die konkrete Identifikation von Barrieren rund um die Krisensituation. Es ist auch anzumerken, dass der Grad der Forscherbeteiligung natürlich über die Zyklen hinweg variiert. Wie bereits erwähnt, werden ab Zyklus 2 Verbesserungen in der Forscherbeteiligung durch eine Reihe von Techniken erreicht, die eine gezieltere und effektivere Prozessautomatisierung mittels RPA in Stresssituationen ermöglicht.

Dies wiederum führt ab Zyklus 2 zu Änderungen in den Praktiken, die es den Forschern ermöglicht, aktiver als zuvor an der RPA-Entwicklung teilzunehmen. Gleichzeitig partizipieren die Forscher, die dann auch autarke Entwicklerrollen von RPA-Lösungen übernehmen, somit auf einer tieferen Ebene und auf eine viel authentischere Weise.

Auf Basis der hier vorgestellten multimodalen Untersuchungen lassen sich Handlungsempfehlungen für die Praxis ableiten. So zeigt sich beispielsweise, dass die vorgestellten Entwicklungsansätze in Form einer RPA-Lösung zunächst auf die infrastrukturellen Bereiche der IT-Architektur bzw. Systemlandschaft priorisiert werden sollten. Entsprechend ist dies nach unserer Beobachtung der entscheidende Faktor für die erfolgreiche Umsetzung einer zeitkritischen, skalierbaren Prozessautomatisierung mit möglichst wenig Konfigurationsaufwand in der besonders heterogenen, schnell wachsenden IT-Systemlandschaft der Behörde.

Wie beschrieben, sorgt die RPA-basierte Übersetzungsschicht dabei für eine minimalinvasive Homogenisierung bestehender und nicht standartisierter Systemstrukturen und Umgebungsvariablen. Damit lassen sich einheitliche Voraussetzungen realisieren, die die fehleranfälligen, ressourcen- und zeitintensiven Entwicklungsprozesse der Parametrisierung von RPA-Lösungen in unserer Betrachtung vermeiden. Hierbei können sichtlich auch die komplexen Anpassungszyklen der RPA-Lösungen effektiv vermieden oder reduziert werden.

Natürlich sind solche vorgestellten Lösungen in der Praxis nicht als dauerhaft anzusehen und sollten vor dem Hintergrund der jeweiligen Situation, in der sich die untersuchte Behörde befand, bewertet und kontextualisiert werden. Unser Artikel gibt jedoch Aufschluss darüber, inwieweit, mit welchem Erfolg und warum es sinnvoll ist, zunächst infrastrukturelle Prozesse, wie z. B. die Anpassung der Systemumgebung, durch RPA zu implementieren – statt standardmäßig nur Geschäftsprozesse für die Automatisierung in Betracht zu ziehen.

Demnach ist die Anwendung der vorgeschlagenen Ansätze für eine beschleunigte Entwicklung von RPA-Lösungen zurzeit noch limitiert. Die vorgestellte Aktionsforschung begrenzt sich auf lediglich eine Gesundheitsbehörde einer ausgewählten Kommune. Obwohl nur eine Behörde untersucht wurde, sind die Ergebnisse aufgrund der vergleichbaren Strukturen im öffentlichen Sektor auch auf viele andere Behördenprofile und andere Kommunen übertragbar. Um die aktuelle besondere Situation der IT-Landschaften der untersuchten Behörde richtig zu beurteilen, muss der Kontext der Betriebsbedingungen während der Pandemie berücksichtigt werden. Der öffentliche Sektor und die Pandemie sind durch sehr spezielle Rahmenbedingungen definiert, die im Normalfall nicht als gegeben angenommen werden. Zum einen existieren viele heterogene Systeme und IT-Strukturen sowie eine nicht übliche Heterogenität des Wissenstandes der eingesetzten Mitarbeiter aufgrund der vorherrschenden Pandemie, die unter anderem komplett neu, fachfremd oder auch fluktierend eingesetzt werden. Diese Gegebenheiten müssen durch die Pandemie zunächst als gegeben hingenommen werden. Kennzeichnend für die Arbeitssituation und die abgeleiteten Ansätze während der Pandemie ist, dass bewusst Insellösungen in Kauf genommen werden, um eine schnell einsetzbare und lauffähige IT-Landschaft bereitzustellen. Aufgrund der bisher anhaltenden Auslastungssituation sind Bemühungen zur Konsolidierung dieser IT-Situation noch nicht vorhanden. Viele neu hinzugekommene IT-Arbeitsplätze bestehen nach wie vor aus heterogenen Komponenten, die nur sehr aufwendig und umständlich betrieben und oft gar nicht konsolidiert werden können. Hinzu kommen weiter wachsende Strukturen, die eine Vereinheitlichung in der aktuellen Situation nicht möglich machen.

Darüber hinaus ist die Objektivität der Ergebnisse eingeschränkt. Die vorgestellten Ansätze beruhen auf Daten, die nicht quantitativ erfasst wurden und unterliegen daher naturgemäß einem gewissen Grad an Subjektivität (Kock 2004). An der hier angewandten Methodik der Aktionsforschung wird aber auch kritisiert, dass die Subjektivität durch den beteiligten Forscher und die damit möglicherweise gegebene Voreingenommenheit bei der Analyse der Ergebnisse zusätzlich verstärkt werden kann. Der zyklische Forschungsprozess in der Aktionsforschung ist auf Verstehen und Handeln ausgerichtet, so dass immer eine Abhängigkeit zwischen den beteiligten Mitarbeitern und den Ergebnissen besteht. Dementsprechend sind die vorgestellten Ansätze zwar in der Praxis entwickelt, eine Verifizierung oder gar Reproduktion der Ergebnisse in anderen Kontexten steht aber noch aus.

Die gegebene Situation und Rahmenbedingungen bringen bereits zuvor dargestellte Limitationen und Besonderheiten mit sich. Um diese Gegebenheiten vor dem Hintergrund des Theoriebeitrages richtig zu beurteilen, muss der Kontext der Einsatzbedingungen während der Pandemie berücksichtigt werden. Die spezifischen Bedingungen verstärkt durch die Pandemie – Heterogenität des Wissensstandes von eingesetztem Personal und die steigende Heterogenität der Systeme und IT-Strukturen beispielsweise bedingt durch neue Arbeitsplätze und Laptops – sowie die bestehenden heterogenen IT-Landschaften in der Behörde, müssen daher akzeptiert werden. Wie bereits erwähnt, werden bewusst Insellösungen in Kauf genommen, um eine schnell einsetzbare und lauffähige IT-Landschaft bereitzustellen.

Nichtsdestotrotz liefert dieser Artikel die Erkentnnis, dass RPA, entgegen dem eigentlichen geforderten Zweck und der bisherigen Forschung sowie wissenschaftlichen Betrachtung, nicht nur Geschäftsprozesse (vertikal) automatisieren kann. Wir zeigen, dass der Einsatz von RPA zudem Infrastrukturen sinnvoll optimieren und einen Mehrwert in der (horizontalen) Systemoptimierung liefern kann. Somit liefert dieser Artikel einen neuen Ansatzpunkt zur bestehenden RPA-Forschung und leistet einen essenziellen Theoriebeitrag in Form einer innovativen Anwendung von RPA in einem bisher unerforschten Szenario. Der Artikel kann damit einen Beitrag zum bestehenden Forschungsstrang über den Nutzen und mögliche Einsatzszenarien einer primär nur als Geschäftsprozessautomatisierung klassifizierten Technologie (RPA) leisten. Dementsprechend eröffnet der Beitrag hier den Diskursraum, den möglichen Einsatz von RPA auch für administrative, systembetreibende Tätigkeiten weiter zu reflektieren.

Vor diesem Hintergrund bietet unser Artikel erste Einblicke, wie RPA mit sehr schnell wachsender und hoher architektonischer IT-Komplexität umgeht, widerspricht im Ergebnis aber der allgemeinen Vorstellung, eine standardisierte und homogenisierte IT-Systemlandschaft in einer Organisation zu erreichen (Leal et al. 2019). Es zeigt sich, dass in heterogenen Systemlandschaften durch den Einsatz von systemunterstützenden RPA-Lösungen sichtbare Stabilitäts- und Geschwindigkeitsgewinne erzielt werden können. Es ist jedoch zu erwarten, dass der tatsächliche Nutzen dieser Lösungen umso größer sein wird, je schneller und unkontrollierter die Softwarelandschaft wächst und je komplexer und unüberschaubarer diese damit wird.

Zukünftige Forschungen sollten durchgeführt werden, um die genauen quantitativen Auswirkungen der vorgestellten Ansätze auf den Projekterfolg nachzuweisen und quantifizierbar zu bestimmen. Um eine weitere Verallgemeinerung zu gewährleisten, müssen Evaluierungen in anderen organisatorischen Hintergründen, auch über längere Zeiträume, durchgeführt werden. Insbesondere sollte weiter untersucht werden, wie sich die Umstände, die Bedingungen und der Kontext der Pandemie im öffentlichen Sektor auf die hier gezeigten Ergebnisse ausgewirkt haben könnten.
